# Climate change, crop production and child under nutrition in Ethiopia; a longitudinal panel study

**DOI:** 10.1186/1471-2458-14-884

**Published:** 2014-08-27

**Authors:** Seifu Hagos, Torleif Lunde, Damen H Mariam, Tassew Woldehanna, Bernt Lindtjørn

**Affiliations:** School of Public health, College of Health sciences, Addis Ababa University, Addis Ababa, Ethiopia; Department of Economics, College of Business and economics, Addis Ababa University, Addis Ababa, Ethiopia; Center for International Health, University of Bergen, Bergen, Norway

**Keywords:** Temperature, Rainfall, Climate change, Under nutrition

## Abstract

**Background:**

The amount and distribution of rainfall and temperature influences household food availability, thus increasing the risk of child under nutrition. However, few studies examined the local spatial variability and the impact of temperature and rainfall on child under nutrition at a smaller scale (resolution). We conducted this study to evaluate the effect of weather variables on child under nutrition and the variations in effects across the three agro ecologies of Ethiopia.

**Methods:**

A longitudinal panel study was conducted. We used crop productions (cereals and oilseeds), livestock, monthly rainfall and temperature, and child under nutrition data for the period of 1996, 1998, 2000 and 2004. We applied panel regression fixed effects model.

**Results:**

The study included 43 clusters (administrative zones) and 145 observations. We observed a spatio temporal variability of rainfall, stunting and underweight. We estimated that for a given zone, one standard deviation increase in rainfall leads to 0.242 standard deviations increase in moderate stunting. Additionally, a one standard deviation increase temperature leads to 0.216 standard deviations decrease in moderate stunting. However, wasting was found to be poorly related with rainfall and temperature. But severe wasting showed a positive relationship with the quadratic term of rainfall.

**Conclusions:**

We conclude that rainfall and temperature are partly predicting the variation in child stunting and underweight. Models vary in predicting stunting and underweight across the three agro ecologic zones. This could indicate that a single model for the three agro ecologies may not be not applicable.

## Background

Ethiopia has experienced repeated famine since the 9^th^ century [1]. Drought due to failure of rains often precedes Ethiopian famines. The failure of rain results in crop failure, impact food productions and usually results in food shortages in vulnerable parts of the population. Historical accounts showed that famine declines after the arrival of rains [2]. Rainfall is hence, one of the most important factors influencing livelihoods of subsistence farmers and pastoralists. Failures or irregularities of the rainy season have a direct link to reduced household food availability [3]. Therefore, in some parts of the country the pattern of rainfall during the main growing season of June-July-August-September (JJAS) has grave consequences on crop availability and child nutrition.

The spatio temporal distribution as well as the amount of rain and temperature influence human health [4]. The influence is substantial in developing countries, such as Ethiopia, which are largely dependent on rain fed agriculture [5]. Climate change impacts food security through multiple pathways. These include altering the availability of food that depends on the agricultural production [6] and influencing the stability of food supplies due to extreme weather events. Moreover, climate impacts are observed through influencing access to food and utilization [7].

There is a marked improvement in children’s anthropometric status in Ethiopia over the past 10 years, as seen by the downward trend in the proportion of children stunted and underweight over the three successive Ethiopian Demographic and Health Survey (EDHS) [8–10]. Although the trend is showing a decreasing pattern, the proportion of under-five stunting, underweight and wasting are still high and more efforts are needed to reach the MDG goals [11]. Climate change is one of the challenges against the efforts undergoing to combat child under nutrition through improved household food security. Ethiopia recently (1999–2000) experienced the effect of low and untimely rainfall [12].

Children are vulnerable to the effects of climate change and examples of these effects are reviewed and reported [13–18]. However, few studies examined the local spatial variability and the impact of climate on stunting, underweight and wasting of under-five children despite the fact that children are considered vulnerable. The purpose of this study was to: (i) evaluate the spatial distribution of rainfall, temperature, per capita crop availability and under nutrition; (ii) characterize the pattern and interrelationship of rainfall, temperature and under nutrition; and (iii) analyze the variations in effects across different agro ecological zones of Ethiopia.

Hence this paper builds on the links between climate variables and under-five children under nutrition and could be used to design appropriate programs for areas impacted by climate change.

## Methods

### Study design and period

We employed a longitudinal panel study design to estimate the effect of growing season temperature and rainfall on child under nutrition for the period of 1996–2004.

### Data and data sources

The dataset constituted a panel of observations of multiple variables. Crop productions (cereals and oil seeds) and livestock data for administrative zones included in the study were obtained from the Central Statistics Agency (CSA Ethiopia) for the period of 1996, 1998, 2000, and 2004. We converted the total amount of crops produced during the main harvesting season (October to November) in each zone into per capita crop availability. We used projected population for each administrative zone. The projected population of zones was estimated by CSA using the ratio method based on the projected population of each region.

We obtained monthly rainfall data for the months of JJAS for each studied zones from the respective weather station(s). The data were made available by the Ethiopian Malaria Prediction System Research Project and the National Meteorological Authority. The main harvest season in most of the study locations is during the months of October and November. We assumed that crop yield is predominantly affected by the amount of rain during the growing seasons of JJAS. Hence we computed the total amount of rainfall for the pre harvest seasons of JJAS and used in this analysis.

The main outcomes of interest for this study were both moderate and severe forms of stunting, wasting, and underweight in children under five years of age. Children were considered moderately malnourished if one of the three forms of under nutrition are 2 SDs (standard deviations) below the median expected height-for-age, weight-for-height and weight-for-age. Children were considered severely malnourished if one of the three forms of under nutrition is 3 SDs below the median expected height-for-age, weight-for-height and weight-for-age.

We used data sets of Agricultural Sample and the Demographic Health Survey (DHS) Surveys collected by the Central Statistics Agency (CSA) that cover all Ethiopian administrative zones from 1996 to 2004. Out of these successive data sets, we created a pseudo panel data set of under nutrition, crop, livestock and other variables at zonal level. The zonal level panel data sets of under nutrition, crop, livestock and other data were matched by year.

Altitude data at 30 arc-seconds (~1 km) resolution data were downloaded by a tile from the world climate data source website (http://www.worldclim.org/bioclim.). The altitudes were then extracted from the raster data set for each study zone. Temperature data were obtained from the Climatic Research Unit (CRU) TS (time-series) datasets (CRU v321) available at http://badc.nerc.ac.uk/view/badc.nerc.ac.uk__ATOM__ACTIVITY_0c08abfc-f2d5-11e2-a948-00163e251233.

### Data processing and analysis

We used Stata (version 11, Stata Corporation, College Station, TX) for panel data analysis. Spatial visualization, extraction of altitude data and mapping was done using Arc GIS version 10 (ESRI).

We applied panel data regression techniques and used the variables per capita crop arability, livestock, rainfall (both linear and quadratic) and temperature in the model to estimate the effect of climate variability on child wasting, underweight and stunting.

Before applying regression, multiple steps were followed. We took logs of crop per capital availability to achieve normality. Based on altitude, we classified the study areas (administrative zones) into three agro ecological zones. A separate model was fit to see the variations in response to climate across the three agro ecological zones. We calculated standardized anomalies for all the variables and used the same in the model. By standardizing we ask if wetter/warmer conditions in any zone leads to more/less under nutrition in the same zone.

Hausman test was conducted in order to choose between fixed or random effects models. The test basically examines whether the error terms are correlated with the regressors. The null hypothesis was that the preferred model is a random effect, while the alternative is the fixed effects. If the error terms are correlated with the one or more of the regressors (such as rainfall), the estimated coefficients are biased and hence the preferred model is the fixed effects.

We used both the linear and quadratic terms for standardized rainfall assuming that stunting, underweight and wasting could be worsened by extreme low and high rainfall. However, we did not check for serial correlation of the residuals as the data set constructed had a shorter timer series.

### Goodness of fits of models

We reported three types of R-square values for each regression model. These are the within, between, and overall R-squares. The within R-square value indicates how much of the variation in child under nutrition with in a zone over the study period is explained by weather variables. The between R-square values indicates how much of the variation in child under nutrition between zones is explained by weather variables. The overall R-square values indicates how much of the overall variation in child under nutrition is explained by climatic variables.

### Non-technical summary of the methods

We constructed a panel dataset using the following steps. For each study zone, data on rainfall, temperature, per capita crop availability and livestock was compiled for the years 1996, 1998, 2000 and 2004. We then matched child under nutrition estimates of each zone with the respective study year. This made a panel data set consisting of a total of 145 observations. A panel data set consists of a cross sectional time series data in which attributes (e.g. Rainfall) of many units (e.g., Zones) are observed over time (e.g. Years). From this data we computed standardized anomalies in order to capture the effects of climate within a given zone. The data were then analyzed using a fixed effects model. We chose the fixed effects model over random effects because each study zone can have a peculiar feature or characteristics that can prevent (e.g. higher productivity) or worsens child nutrition condition and this must be accounted in the analysis.

## Results

### Sample characteristics

The study included 43 clusters (administrative zones) and 145 observations for the period of 1996–2004. This period was one of the recent periods that Ethiopia experienced the effect of low and untimely rainfall [12]. There were on average 3.5 observations per cluster (zone) in the data set. A descriptive summary of the panel data set used for the present analysis is presented (Table 1).

**Table 1:** **Summary of panel data used in the study, Ethiopia, 1996–2004**

Variable		Mean	Std. dev.	Min	Max	Observations*
Rainfall (mm)	Overall	645.2	317.4	41.2	1377.9	N = 145
	Between		306.5	64.4	1225.4	n = 41
	Within		122.3	258.8	1055.0	T-bar = 3.5
Temperature (°C)	Overall	19.92	3.12	15.5	29.9	N = 142
	Between		3.56	15.8	29.9	n = 41
	Within		0.19	19.44	20.3	T-bar = 3.6
Per capita crop (kg)	Overall	206.9	166.9	10.8	1022.3	N = 127
	Between		123.6	22.9	528.6	n = 34
	Within		115.5	-106.5	872.6	T-bar = 3.73529
Wasting (%)	Overall	10.1	3.7	2.8	24.6	N = 145
	Between		2.9	3.4	17.9	n = 41
	Within		2.6	2.7	16.8	T-bar = 3.5
Severe wasting (%)	Overall	3.3	2.1	0.0	16.3	N = 143
	Between		1.5	0.0	9.2	n = 41
	Within		1.6	-0.7	10.4	T-bar = 3.4
Underweight (%)	Overall	42.5	9.5	19.0	62.5	N = 145
	Between		7.4	27.6	57.9	n = 41
	Within		6.3	23.5	57.7	T-bar = 3.5
Severe underweight (%)	Overall	15.4	5.6	3.4	30.3	N = 145
	Between		4.1	7.5	21.8	n = 41
	Within		4.0	5.2	24.9	T-bar = 3.5
Stunting (%)	Overall	55.2	11.2	20.4	78.6	N = 145
	Between		7.4	38.3	70.6	n = 41
	Within		8.8	28.2	73.8	T-bar = 3.5
Severe stunting	Overall	32.8	10.0	8.0	54.2	N = 145
	Between		6.8	18.5	45.9	n = 41
	Within		7.5	15.4	50.9	T-bar = 3.5

*N = total number of observations, n = the number of clusters (zones), T-bar = average observation per cluster (zone).

### Summary of the model parameters

The average growing season rainfall of the study locations was 645.2 mm. The overall amount of growing season rainfall ranges from 41.2 to 1378 mm. The average minimum and maximum amount of rainfall with in zones was 64.4 and 1225.4 mm respectively. However, the average minimum and maximum amount of rainfall documented between zones was 258 and 1055 mm respectively. We observed a comparatively higher variability of rainfall between zones compared to within zones.

We found an average growing season temperature of 19.9°C for the study period. The overall minimum and maximum temperatures for the study locations were 15.5°C and 29.9°C respectively. Unlike rainfall, we observed a smaller variability of temperature within zones over the study period.

Low amount of rainfall during the growing season is expected to affect crop production and availability. Data on total per capita crop availability in the studied zones indicated that the overall mean total per capita crop availability during the study period was 206.9 kg. Per capita crop availability in the study ranged from 10.8 kg to 1022.3 kg.

The overall average prevalence of underweight over the study period was 42.5% (range: 19, 62.5%). About 15.4% had a severe degree of underweight. The overall average stunting during the study period was 55.2% (range: 20.4, 78.6%). About 32.8% of the children had severe stunting. The mean wasting prevalence over the study period was 10.1%. We observed a higher variability in the prevalence of moderate stunting, wasting and underweight. The variability was consistent between as well as within the zones over the study period.

### Spatial and temporal pattern

The average growing season rainfall shows marked variation over the study period as well as agro-ecologies. The average growing season rainfall showed a decreasing pattern between the highlands to the lowlands. The highlands had an average rainfall of 726.8 mm, while the midlands and lowlands had 668.0 mm and 513.9 mm respectively. The average growing season temperature didn’t show a marked variation over the study period. We observed a relatively higher temperature in lowland compared to the midlands and highlands (Table 2).

**Table 2:** **Distribution of rainfall, temperature, per capita crop and child under nutrition status by agro ecologies and study period, Ethiopia, 1996–2004**

Agro ecology	Year	Rainfall (mm) (mean, SD)	Temperature (°C) (mean, SD)	Per capita crop (kg)	wasting (%) (mean, SD)		Underweight (%) (mean, SD)		Stunting (%) (mean, SD)	
					Moderate	Severe	Moderate	Severe	Moderate	Severe
**Lowland**	1996	547.3(335.9)	21.42.0)	391.1(210.5)	8.1(2.7)	3.9(0.7)	41.6(7.9)	16.7(5.3)	63.1(6.3)	42.4(8.3)
	1998	540.8(401.3)	23.1(3.0)	130.6(121.8)	9.5(3.8)	2.9(1.8)	41.0(8.0)	14.0(4.9)	55.8(7.6)	32.2(6.5)
	2000	512.0(414.7)	23.6(3.4)	162.5(154.8)	11.2(4.5)	3.7(3.9)	38.4(9.8)	13.9(6.6)	47.6(9.7)	27.0(8.0)
	2004	464.6(374.0)	22.5(2.3)	138.2(136.3)	9.8(5.4)	2.8(2.5)	34.7(10.1)	12.4(6.9)	41.7(10.5)	21.3(7.9)
	Total	513.9(377.2)	22.8(2.6)	202.3(185.1)	9.9(4.3)	3.3(2.6)	38.79(9.2)	14.0(6.0)	51.0(11.5)	29.6(10.3)
**Midlands**										
	1996	721.1(165.4)	18.5(2.3)	259.4(129.1)	9.7(2.6)	4,2(1.1)	43.0(6.3)	17.9(4.6)	65.0(6.0)	43.7(6.8)
	1998	751.8(304.1)	19.7(2.7)	187.3(197.6)	11.6(4.4)	2.9(1.4)	48.4(8.0)	16.3(4.9)	57.3(7.4)	32.8(7.2)
	2000	646.4(263.5)	19.5(2.9)	224.8(167.3)	12.0(2.5)	4.7(2.2)	45.7(10.7)	18.6(5.7)	56.8(7.6)	33.4(7.3)
	2004	645.3(292.9)	19.7(2.8)	278.2(271.8)	10.8(4.2)	3.1(1.8)	35.7(4.6)	10.3(3.1)	43.9(10.9)	22.2(7.7)
	Total	688.0(281.9)	19.4(2.7)	233.9(198.0)	11.2(3.6)	3.7(1.9)	43.2(9.1)	15.6(5.7)	54.8(11.0)	32.2(9.8)
**Highlands**										
	1996	733.0(165.4)	17.5(1.4)	217.1(160.6)	9.6(2.5)	4.1(1.5)	45.9(7.6)	19.5(5.3)	64.7(6.4)	43.9(7.4)
	1998	744.2(194.5)	18.1(1.5)	163.5(111.9)	9.9(3.3)	2.3(1.0)	49.3(8.0)	15.3(4.1)	62.3(8.7)	35.0(7.3)
	2000	739.1(340.3)	17.9(1.6)	186.9(100.2)	10.5(2.7)	3.3(1.5)	38.4(9.8)	18.9(3.6)	61.8(6.7)	37.8(6.80
	2004	691.2(274.0)	18.1(1.5)	182.2(271.8)	7.0(2.6)	2.1(1.2)	36.1(7.5)	12.8(3.7)	49.5(9.4)	28.6(7.8)
	Total	726.8(247.3)	17.9(1.5)	186.99118.0)	9.26(3.0)	2.9(1.5)	45.3(9.1)	16.6(4.9)	59.5(9.8)	36.2(9.0)
**All zones**		645.2(317,4)	19.92(3.11)	206.9(167.0)	10.1(3.7)	3.3(2.1)	42.5(9.5)	15.4(5.6)	55.2(11.2)	32.8(10.0)

We observed a decreasing pattern on stunting, wasting and underweight over the study periods. The highlands and midlands documented a relatively higher prevalence of stunting and underweight compared to the lowlands. The average prevalence of underweight and stunting among highlands was 45.3 and 59.5% respectively, while the average prevalence of underweight and stunting among lowlands was 38.7 and 51.0% respectively. However, the prevalence of severe forms of stunting, underweight and wasting did not vary over the three agro ecologic zones (Table 2).

**Figure 1:**
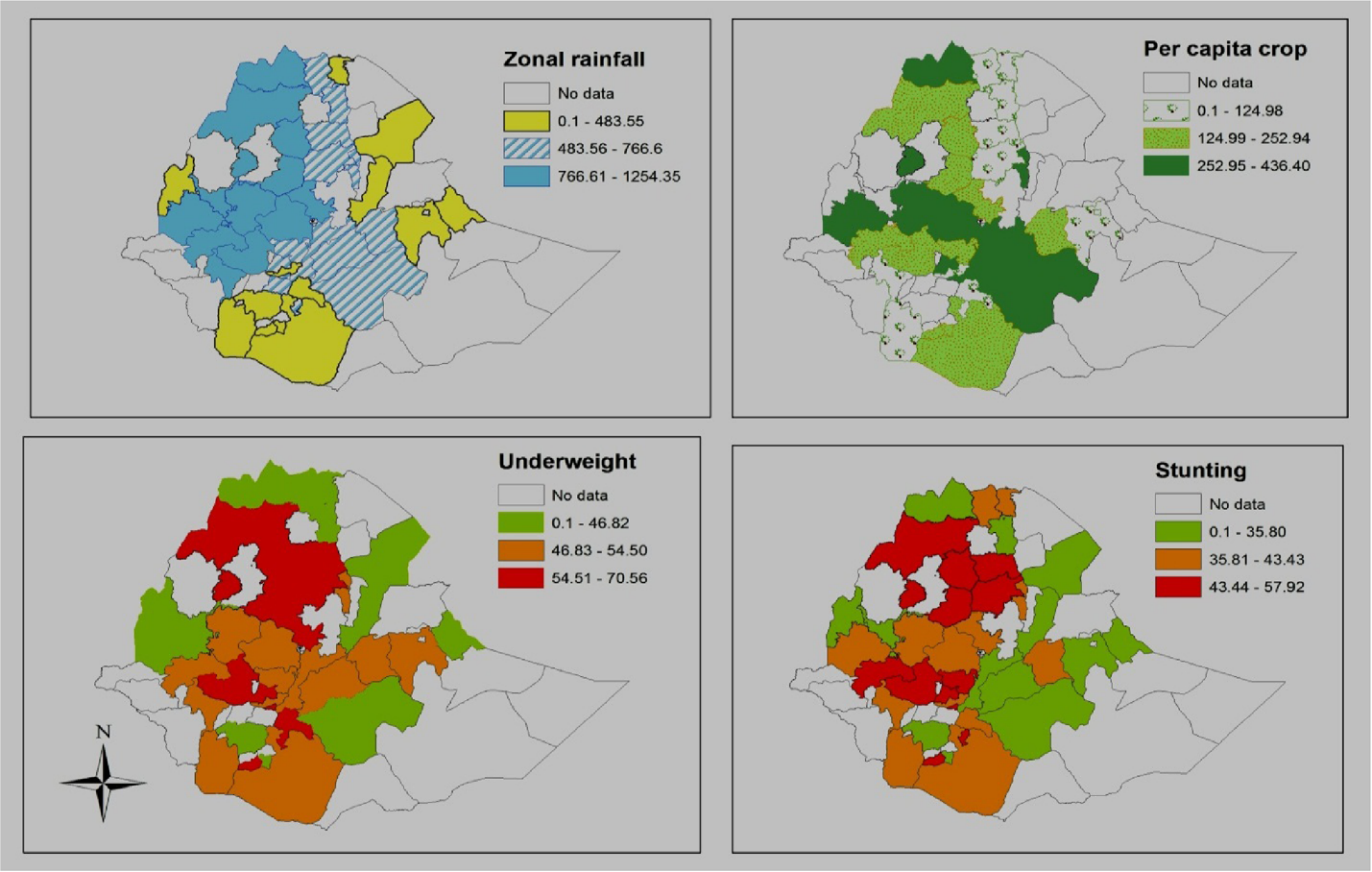
**Choropleth maps showing spatial distribution of rainfall, per capita crop availability and under nutrition for the study locations, Ethiopia,1996-2004.**

Figure 1 shows the spatial pattern of average growing season rainfall, per capita crop availability, stunting and underweight in the study zones. The choropleth map indicates zones in the north and northwestern part of the country documented higher rainfall. Higher per capita crop availability is documented in some zones with high rainfall. We noted that zones with higher rainfall documented a higher prevalence of stunting and underweight compared with zones with lower rainfall. However, zones with higher per capita crop availability had a relatively lower prevalence of child under nutrition rates.

### Panel regression results

We observed the following relationships from the panel regression models for moderate and severe stunting (Tables 3 and 4). First, stunting was found to be strongly and negatively correlated with growing season temperature for the three agro ecologies. For a given zone, one standard deviation increase in temperature resulted in 0.216 standard deviation decrease in moderate stunting. This relationship is statistically significant for the all zone and the lowland models. Second, stunting is positively associated with the amount of rainfall, indicating that an increase in rainfall resulting in an increas. For a given zone, one standard deviation increase in rainfall resulted in 0.242 standard deviation increase in moderate stunting. This relationship is statistically significant for the all zone and midlands model. We did not find any significant result on the relationship between rainfall and stunting when the quadratic terms instead of the linear form of rainfall was used. However, the direction of the coefficients indicates that extreme forms rainfall is leading to a higher prevalence of moderate stunting. Similar results were documented on the relationship between severe form of stunting and weather variables.

**Table 3:** **Panel regression results on the effect of rainfall and temperature on stunting, Ethiopia, 1996–2004**

	All zone model		Lowlands		Midlands		Highlands	
	coef†	se‡	coef	se	coef	se	coef	se
Rainfall during growing season	0.242***	0.91	0.06	0.17	0.495**	0.16	0.22	0.16
Temperature	-0.216*	0.12	-0.55**	0.26	-0.09	0.17	-0.23	0.22
Quadratic term for rainfall	0.060	0.14	-0.33	0.27	0.30	0.22	0.23	0.27
Per capita crop	-0.53	0.000	0.003	0.18	0.12	0.16	-0.000	0.17
Livestock	-0.02*	0.068	-0.27*	0.24	-0.52	0.21**	0.06	0.18
Number of observations	121		32		39		50	
Within R^2^	0.28		0.53		0.49		019	
Between R^2^	0.81		0.07		0.95		0.87	
Overall R^2^	0.30		0.53		0.5		0.19	

Note: ***p < 0.01, **p < 0.05, *p < 0.1 , †model coefficients, ‡standard errors of the coefficient.

**Table 4:** **Panel regression results on the effect of rainfall and temperature on severe stunting, Ethiopia, 1996–2004**

	All zone model		Lowlands		Midlands		Highlands	
	coef†	se‡	coef	se	coef	se	coef	se
Rainfall during growing season	0.225***	0.81	-0.01	0.17	0.51**	0.16	0.17	0.13
Temperature	-0.24*	0.11	-0.64**	0.26	-0.28	0.18	-0.13	0.17
Quadratic term for rainfall	0.52	0.12	-0.39	0.27	0.26	0.22	0.27	0.21
Per capita crop	0.13	0.85	0.99	0.18	0.23	0.16	0.13	0.13
Livestock	-0.39***	0.10	-0.15	0.22	-0.36	0.21**	-0.46***	0.15
Number of observations	121		32		39		50	
Within R^2^	0.43		0.56		0.50		0.47	
Between R^2^	0.82		0.03		0.81		0.97	
Overall R^2^	0.44		0.56		0.51		0.48	

Note: ***p < 0.01, **p < 0.05, *p < 0.1 , †model coefficients, ‡standard errors of the coefficient.

Tables 5 and 6 shows results from the models where moderate and severe underweight is regressed on growing season rainfall and temperature. The results across the agro ecological zones consistently showed inverse relationships between growing season temperature and moderate form of underweight, although the relationship was not statistically significant. A severe form of underweight, however, showed a statistically significant inverse association with temperature. We observed that the quadratic term for rainfall is significantly related with underweight in the highland models indicating that a very small as well as high amount of rainfall leading to higher prevalence of underweight. This relation might indicate a nonlinear relationship between rainfall and underweight in the highlands of Ethiopia.

**Table 5:** **Panel regression results on the effect of rainfall and temperature on underweight, Ethiopia, 1996–2004**

	All zone model		Lowlands		Midlands		Highlands	
	coef†	se‡	coef	se	coef	se	coef	se
Rainfall during growing season	0.19*	0.10	-0.17	0.21	0.36*	0.20	0.23	0.16
Temperature	-0.12	0.14	-0.29	0.34	0.07	0.23	-0.21	0.23
Quadratic term for rainfall	0.12	0.16	-0.32	0.35	0.21	0.27	0.49*	0.26
Per capita crop	-0.07	0.11	0.10	0.23	-0.21	0.20	-0.05	0.16
Livestock	-0.08	0.12	-0.16	0.28	-0.31	0.24	0.15	0.18
Number of observations	121		32		39		50	
Within R^2^	0.09		0.22		0.22		0.25	
Between R^2^	0.80		0.03		0.45		0.25	
Overall R^2^	0.09		0.22		0.22		0.25	

Note: *p < 0.1 , †model coefficients, ‡standard errors of the coefficient.

**Table 6:** **Panel regression results on the effect of rainfall and temperature on severe underweight, Ethiopia, 1996–2004**

	All zone model		Lowlands		Midlands		Highlands	
	coef†	se‡	coef	se	coef	se	coef	se
Rainfall during growing season	0.14	0.10	-0.27	0.20	0.42**	0.18	0.19	0.15
Temperature	-0.26**	0.13	-0.35	0.30	-0.26	0.21	-0.32	0.17
Quadratic term for rainfall	0.16	0.15	-0.39	0.31	0.02	0.25	-0.03	0.24
Per capita crop	0.17*	0.01	0.37*	0.21	0.14	0.18	0.14	0.15
Livestock	-0.18	0.12	0.09	0.25	-0.27	0.22	-0.27	0.17
Number of observations	121		32		39		50	
Within R^2^	0.24		0.36		0.35		0.35	
Between R^2^	0.06		0.01		0.07		0.97	
Overall R^2^	0.24		0.36		0.34		0.35	

Note: **p < 0.05, *p < 0.1 , †model coefficients, ‡standard errors of the coefficient.

In the present study wasting was found to be poorly related with rainfall and temperature (Table 7). None of the models resulted a significant relationship with rainfall and temperature. But severe wasting showed a positive relationship with the quadratic term of rainfall in the all zone as well as the midland models (Table 8).

**Table 7:** **Panel regression results on the effect of rainfall and temperature on wasting, Ethiopia, 1996–2004**

	All zone model		Lowlands		Midlands		Highlands	
	coef†	se‡	coef	se	coef	se	coef	se
Rainfall during growing season	-0.06	0.10	-0.04	0.17	0.01	0.20	-0.03	0.18
Temperature	-0.14	0.14	0.33	0.26	-0.32	0.24	-0.21	0.23
Quadratic term for rainfall	0.09	0.16	-0.37	0.27	0.31	0.28	0.37	0.29
Per capita crop	-0.04	0.11	0.33	0.18	-0.22	0.20	-0.02	0.18
Livestock	0.11	0.13	0.16	0.22	0.15	0.25	-0.04	0.20
Number of observations	121		32		39		50	
Within R^2^	0.02		0.2		0.50		0.47	
Between R^2^	0.17		0.00		0.81		0.97	
Overall R^2^	0.02		0.2		0.51		0.48	

Note:, †model coefficients, ‡standard errors of the coefficient.

**Table 8:** **Panel regression results on the effect of rainfall and temperature on severe wasting, Ethiopia, 1996–2004**

	All zone model		Lowlands		Midlands		Highlands	
	coef†	se‡	coef	se	coef	se	coef	se
Rainfall during growing season	0.04	0.10	0.09	0.18	0.29	0.20	-0.14	0.15
Temperature	-0.11	0.13	0.05	0.28	-0.17	0.23	-0.11	0.2
Quadratic term for rainfall	0.26*	0.14	0.19	0.29	0.49*	0.26	0.26	0.25
Per capita crop	0.26**	0.1	0.62	0.19**	0.09	0.19	0.23	0.15
Livestock	-0.26**	0.12	-0.10	0.23	-0.32	0.23	-0.41**	0.17
Number of observations	121		32		39		50	
Within R^2^	0.27		0.45		0.22		0.35	
Between R^2^	0.05		0.02		0.45		0.91	
Overall R^2^	0.26		0.45		0.22		0.35	

Note: **p < 0.05, *p < 0.1 , †model coefficients, ‡standard errors of the coefficient.

### Goodness of fits of models

Except for the lowlands, the variation in moderate and severe stunting between study zones is adequately explained by climatic variables in the model (between R square values; 0.81-0.95). However, the variation in stunting with a study zone over the study period is poorly explained by the present model. The R-square values for models on underweight were very small and vary over the three agro ecologies. The overall R-square values indicated that these models capture smaller aspects of the variation in child underweight between as well as within the study zones over the study period.

## Discussion

We used existing data to explore spatio temporal patterns and further to estimate the impact of growing season temperature and rainfall on child underweight, wasting and stunting before, during and after the crisis period of 1999–2000 [12].We found that unlike temperature, rainfall showed a marked variation over the study periods as well as agro-ecologies. Although, there is a decreasing pattern of stunting, wasting and underweight over time , a higher prevalence of stunting and underweight were found in the highlands and midlands compared to the lowlands. We found that the amount and direction of the effect of rainfall vary among the different ecologies. Additionally, sometimes the quadratic terms of rainfall rather than the linear forms were significant predictors for underweight and stunting. Moreover, the results of the study demonstrate that temperature has a significant effect on child underweight and stunting.

The reported prevalence of stunting, wasting, and underweight from this study are relatively higher when compared with successive EDHS [8–10]. However, a similar result was observed with regard to the decreasing trend in stunting and underweight over the study years. Moreover when the prevalence of stunting and underweight of the present study is compared with EDHS survey of the same year, we found a comparable figure All these taken might suggest that the sample could be represent significantly major parts of Ethiopia.

A similar approach was used to provide evidence on the association between climate change and child under nutrition in Mali, Africa. [19]. Stunting is found to be highly influenced by arid climate even when controlled for livelihoods. However, the effect of climate on underweight is found to be not significant. Some argue that underweight is a short term response to climate seasonal flux or shocks and these shocks can be absorbed and modified by livelihood adaptation capabilities. Moreover, sometimes the effects of decline in rainfall (and crop failure) on child hood anthropometries indices may not be visible as it could be prevented through public health measures [20]. Our study also documented that the model for underweight showed non-significant association with rainfall as compared to models for stunting.

The finding that rainfall and temperature predicting child stunting has important implications over future child under nutrition attributable to climate change. A relative increase on moderate and severe forms of stunting is estimated due to climate change in sub-Saharan African countries [21]. However, uncertainties still remain on the pattern of future rainfall in Eastern African countries including Ethiopia. Studies done by Christensen et al. reported a higher probability of an increase in the annual mean rainfall in East Africa extending to the Horn of Africa [22]. The growing seasons of countries such as Ethiopia would be benefited due to a combination of increased rainfall as well as temperature indicating that not all changes in climate variability would be negative [23]. In contrary, Funk et al. [24] indicated that warming of the Indian Ocean would lead to a decrease in rainfall and hence can threaten Eastern Africa. Some argue that the precipitation simulation by IPPC did not consider the complex terrain nature of the Eastern Africa [24].

Assessing the effect of climate variability on health poses methodological challenges. The common challenges include exposure assessment, ecological fallacies, the complexity of relationships, and scale of the study [25, 26]. First, in this study we assumed that the exposure (rainfall and temperature) will be similar for households that are found in the same zone (group) as climate impacts populations rather than individuals [25]. Hence we interpreted the link between rainfall, temperature and child under nutrition at zonal (group) level using aggregated estimates. However, in the absence of individually collected data, it is somehow difficult to exclude totally the role of ecological fallacy in the relationship. Moreover, we cannot rule out the local variation in the exposures such as rainfall and temperature within a given zone. Second, we used the UNICEF conceptual framework [27] to develop a biologically plausible model. Immediate and underlying causes of child under nutrition are captured with model variable such illness prevalence, livestock and per capita crop availability. However, the actual relationship can be more complex than assumed, and can be nonlinear requiring multiple pathways.

The findings of the current study shall be interpreted within the context of the following limitations. Due to the limitation of the availability of complete data for such work, the sample sizes for a stratified analysis based on agro-ecological zones were small. This has likely resulted in the absence of significant results. We were not also able to quantify and characterize threshold limits of rainfall which would have been beneficial to model child under nutrition risks. Despite these limitations, we believe that the present study involved more than half of the administrative zones of Ethiopia and was able to generate important information on the variation in effects of weather variables on child under nutrition.

## Conclusions

We conclude that rainfall and temperature are partly predicting the variation in stunting and underweight in Ethiopia. Moreover, the models vary in predicting stunting and underweight across the three agro ecologic zones. This could indicate that a single model for all the three agro ecologies may not be not applicable. We recommend further work but at a micro level using similar analysis methods to assess the effect of rainfall and temperature on stunting, wasting and underweight.

## References

[1] Taye A, Mariam DH, Murray V (2010). Interim report: review of evidence of the health impact of famine in Ethiopia. Perspect Public Health.

[2] Kloss H, Lindtjorn B (1994). Malnutrition during recent famines in Ethiopia. Northeast Afr Stud.

[3] Haile M (2005). Weather patterns, food security and humanitarian response in sub-Saharan Africa. Philos Trans R Soc Lond B Biol Sci.

[4] Campbell-Lendrum D, Woodruff R (2006). Comparative risk assessment of the burden of disease from climate change. Environ Health Perspect.

[5] Parrya ML, Rosenzweigb C, Iglesiasc A, Livermored M, Fischere G (2004). Effects of climate change on global food production under SRES emissions and socio-economic scenarios. Glob Environ Chang.

[6] Lobell DB, Schlenker W, Costa-Roberts J (2011). Climate trends and global crop production since 1980. Science.

[7] Confalonieri U, Menne B, Akhtar R, Ebi KL, Hauengue M, Kovats RS, Revich B, Woodward A (2007). Human health. Climate Change 2007: Impacts, Adaptation and Vulnerability. Contribution of Working Group II to the Fourth Assessment Report of the Intergovernmental Panel on Climate Change.

[8] Central Statistical Agency [Ethiopia] and ORC Macro (2001). Ethiopia Demographic and Health Survey 2000.

[9] Central Statistical Agency [Ethiopia] and ORC Macro (2006). Ethiopia Demographic and Health Survey 2005.

[10] Central Statistical Agency [Ethiopia] aII (2012). Ethiopia Demographic and Health Survey 2011.

[11] Stevens GA, Finucane MM, Paciorek CJ, Flaxman SR, White RA, Donner AJ, Ezzati M (2012). Trends in mild, moderate, and severe stunting and underweight, and progress towards MDG 1 in 141 developing countries: a systematic analysis of population representative data. Lancet.

[12] Hammond L, Maxwell D (2002). The Ethiopian crisis of 1999–2000: lessons learned, questions unanswered. Disasters.

[13] Bernstein AS, Myers SS (2011). Climate change and children’s health. Curr Opin Pediatr.

[14] Ebi KL, Paulson JA (2007). Climate change and children. Pediatr Clin North Am.

[15] Ebi KL, Paulson JA (2010). Climate change and child health in the United States. Curr Probl Pediatr Adolesc Health Care.

[16] Etzel RA (2010). Climate change and child health in the United States: foreword. Curr Probl Pediatr Adolesc Health Care.

[17] McCartney PR (2007). Climate change and child health. MCN Am J Matern Child Nurs.

[18] Seal A, Vasudevan C (2011). Climate change and child health. Arch Dis Child.

[19] Jankowskaa MM, Lopez-Carrb D, Funkc C, Husakd GJ, Chafee ZA (2011). Climate change and human health: Spatial modeling of water availability, malnutrition, and livelihoods in Mali, Africa. Appl Geogr.

[20] Kumar HR, Venkaiah K, Arlappa N, Kumar S, Brahmam GNV, Vijayaraghavan K (2005). Diet and Nutritional Situation of the Population in the Severely Drought Affected Areas of Gujarat. J Hum Ecol.

[21] Lloyd SJ, Kovats RS, Chalabi Z (2011). Climate change, crop yields, and undernutrition: development of a model to quantify the impact of climate scenarios on child undernutrition. Environ Health Perspect.

[22] Christensen JH, Hewitson B, Busuioc A, Chen A, Gao X, Held I, Jones R, Kolli RK, Kwon W-T, Laprise R, Magaña Rueda V, Mearns L, Menéndez CG, Räisänen J, Rinke A, Sarr A, Whetton P, Solomon S, Qin D, Manning M, Chen Z, Marquis M, Averyt KB, Tignor M, Miller HL (2007). Regional Climate Projections. Climate Change 2007: The Physical Science Basis. Contribution of Working Group I to the Fourth Assessment Report of the Intergovernmental Panel on Climate Change.

[23] Thornton PK, Jones PG, Owiyo TM, Kruska RL, Herero M, Kristjanson P, Notenbaert A, Bekele N, Omolo A (2006). Mapping Climate Vulnerability and Poverty in Africa. Report to the Department for International Development.

[24] Funk C, Dettinger MD, Michaelsen JC, Verdin JP, Brown ME, Barlow M, Hoell A (2008). Warming of the Indian Ocean threatens eastern and southern African food security but could be mitigated by agricultural development. Proc Natl Acad Sci U S A.

[25] Xun WW, Khan AE, Michael E, Vineis P (2010). Climate change epidemiology: methodological challenges. Int J Public Health.

[26] Ebi KL (2008). Healthy people 2100: modeling population health impacts of climate change. Clim Change.

[27] UNICEF (1990). Strategy for Improved Nutrition of Children and Women in Developing Countries: A UNICEF Policy Review.

[CR28] The pre-publication history for this paper can be accessed here:http://www.biomedcentral.com/1471-2458/14/884/prepub

